# A high‐salt diet induces synaptic loss and memory impairment via gut microbiota and butyrate in mice

**DOI:** 10.1002/imt2.97

**Published:** 2023-03-21

**Authors:** Chao Lei, Cong Liu, Yuling Peng, Yu Zhan, Xiaoming Zhang, Ting Liu, Zhihua Liu

**Affiliations:** ^1^ Department of Anorectal Surgery, Affiliated Dongguan Hospital Southern Medical University (Dongguan People's Hospital) Dongguan China; ^2^ Innovation Centre for Advanced Interdisciplinary Medicine, Key Laboratory of Biological Targeting Diagnosis, Therapy and Rehabilitation of Guangdong Higher Education Institutes The Fifth Affiliated Hospital of Guangzhou Medical University Guangzhou China; ^3^ Department of Internal Medicine Huazhong University of Science and Technology Union Shenzhen Hospital Shenzhen China

## Abstract

High‐salt diet (HSD)‐fed mice display cognitive impairment and lower synaptic proteins via changed gut microbiota composition and short‐chain fatty acids production. Gut microbiota from HSD‐fed mice impairs memory and synapse in normal salt diet‐fed mice. Butyrate treatment partially reverses memory impairment in HSD‐fed mice. Above all, this study indicates the important role of the gut microbiome and butyrate production in synaptic loss and memory impairment.

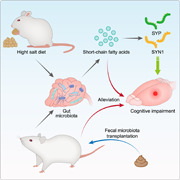

Excess salt intake impairs memory function in mice models but its significance on the gut microbiota remains largely unexplored. In this study, we explored the relationship among the gut microbiota, high‐salt diet (HSD), synapses, and memory function. The Y‐maze test and novel object recognition task were performed to evaluate short‐term and long‐term memory, respectively. Real‐time quantitative polymerase chain reaction (PCR) and western blot analysis were conducted to evaluate the expression level of synaptic proteins. In addition, microbiome 16S ribosomal DNA (rDNA) sequencing was selected to evaluate changes in fecal microbiota, short‐chain fatty acids were detected by gas chromatography–mass spectrometry (GC‐MS) analysis and brain gene changes were determined by transcriptome sequencing. The results showed that HSD affected the relative abundance of butyrate‐producing bacteria (*Butyricimonas virosa*) and butyrate production, which consequently decreased the expression of synaptic proteins (SYN1) and impaired short‐ and long‐term memory. Similar results were observed following fecal microbiota transplantation (FMT). Further, the effects of HSD on memory impairment were related to the phosphoinositide 3‐kinase (PI3K)/protein kinase B (Akt) pathway. This study revealed that HSD affected the gut microbiota and butyrate production, which influenced synapses and memory through the synaptic protein SYN1 via the PI3K‐Akt pathway.

The decline in cognitive health is rising due to higher and higher life expectancy [1] and unhealthy dietary habits [2], among which a HSD is associated with cognitive impairment [3]. Memory functions are the first core cognitive areas to be compromised [4]. Although mice fed with HSD demonstrated memory deficits with synaptic protein deregulation [5], the molecular mechanisms remain unknown.

Memory impairment is usually caused by various neurological disorders [6]. The microbiota–gut–brain axis is pivotal in regulating cognitive functions. In addition, there is growing evidence supporting the role of microbiota in cognitive impairment [7, 8] and specific microbiota in memory functions. For instance, it is reported that *Lactobacillus helveticus* could prevent memory impairment induced by western diets [9]. The gut microbiota was also reported to impact neurodevelopment, synaptic plasticity, myelination, and complex host behaviors through various pathways [10], including the short‐chain fatty acids (SCFA) metabolism [11]. Dietary pattern is a key modulator of gut microbiota composition that affects human health and biological processes [12, 13] and HSD can alter the mutualism between host and gut microbiota [14]. It was reported that sodium butyrate (NaB) could improve radiation‐induced cognitive injury through the pathway of hippocampal phosphorylated cAMP response element binding protein [15]. Another study showed that NaB could ameliorate synaptic plasticity in mice [16]. The intraperitoneal administration of NaB was shown to immediately cause memory reactivation and enhanced memory in rats with weak memory [17]. However, it remains unclear whether HSD‐induced microbiota dysbiosis and cognitive impairment have a correlative relationship or whether microbiota dysbiosis mediates HSD‐induced cognitive impairment.

The PI3K/Akt pathway is an essential mediator of cell growth, survival, and differentiation in neurons, which involves cognitive impairment and synapse formation and maintenance [18, 19]. The activity of the PI3K‐Akt pathway was found to be fundamental for microtubule transport during axon growth cone formation as the brain develops, which is considered essential for the adult brain because of its role in maintaining synaptic plasticity [20]. This pathway can increase the phosphorylation of GABA receptors and the survival of dentate gyrus granule cells, which are the two processes guaranteed to promote synaptic strength and plasticity [21]. Dysbiosis of the gut microbiota and SCFAs have been implicated as modulators for this pathway [22].

Herein, we hypothesized that synapse function and memory impairment were associated with both HSD and a specific gut microbiota. In our study, we evaluated the memory function, synapse protein expression, gut microbiota composition, and SCFAs production in mice fed with HSD and a normal salt diet (NSD). Furthermore, we also investigated whether FMT from mice fed with HSD into mice fed with NSD could help identify factors that impact the brain's transcriptome and explored the relationship between gut microbiota, diet (HSD or NSD), synapse, and memory.

## RESULTS

### Mice fed HSD displays cognitive impairment and lower synaptic protein

**Figure 1:**
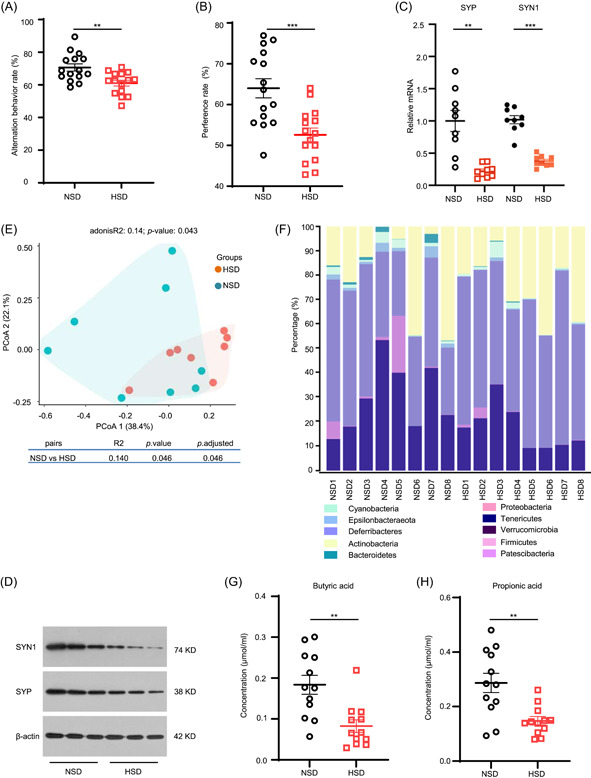
High‐salt diet (HSD) impairs memory and synapse. (A) Percentage of spontaneous alternations in performance in the Y‐maze test. A total of 30 mice were included, with 15 in each group. (B) Percentage of exploration time during recognition trail in the novel object recognition task (*n* = 15/group; total = 30 mice). (C) SYP and SYN1 messenger RNA (mRNA) levels in the hippocampus (*n* = 9/group; total = 18 mice). (D) Western blot analysis confirmed the expression level of SYP and SYN1 (*n* = 9/group; total = 18 mice). (E) Principal component analysis plot (PCoA) based on bacterial 16S ribosomal DNA gene sequencing. (F) Profiles of the top 10 gut microbiota at the level of phylum. The levels of butyric acid (G) and propionic acid (H) measured by gas chromatography–mass spectrometry (GC‐MS) from fecal samples. Data are expressed as mean ± SEM; *p* values were calculated by the nonparametric Mann–Whitney test; *p < 0.05; **p < 0.01; ***p < 0.001.

To explore the effects of HSD on cognitive function, we performed two behavioral tests to evaluate short‐ and long‐term memory function separately. The NSD and HSD‐fed mice exhibited similar food intake, body weight (Supporting Information: Figure 1A,B), and length of small intestine and colon (Supporting Information: Figure 1C,D). However, the HSD‐fed mice had a higher water and salt intake (Supporting Information: Figure 1E,F). Continuous spontaneous alternation behavior was decreased in the HSD group (Figure 1A), indicating short‐term memory impairment. The total entries were slightly higher than that in the NSD group (Supporting Information: Figure 2A), which might have been elicited by HSD‐related anxiety.

Meanwhile, the HSD group displayed defects in long‐term memory, indicated by a decreased ratio of exploration time in the novel object recognition task (Figure 1B). However, there was no difference in total exploration time in the recognition phase (Supporting Information: Figure 2B), which suggested that HSD had no notable effect on locomotion. During the probe trial of the Morris water maze, the number of platform crossings was lower in the HSD group than control (Supporting Information: Figure 3A,B), consistent with previously reported results [23]. Furthermore, compared with the NSD group, the HSD group exhibited decreased SYP and SYN1 messenger RNA (mRNA) levels (Figure 1C,D).

### HSD impairs memory and synapse via gut microbiota composition and SCFAs production

The fecal microbiome was determined by 16S rDNA sequencing (Supporting Information: Figure 4A). No significant differences were found in the Shannon index and Chao1 index (Supporting Information: Figure 4B,C). Moreover, we observed a significant differential clustering of gut microbiota community induced by HSD, which was supported by the Bray–Curtis dissimilarity (Figure 1E) and Jaccard distances (Supporting Information: Figure 4D), and a similar finding was observed by Nonmetric Multidimensional Scaling (NMDS) (Supporting Information: Figure 4E).

To investigate the shift in gut microbiota, we analyzed the abundance of different bacterial phyla between both groups using OTUs (Figure 1F). Compared with mice fed with NSD, we found that the phyla *Bacteroidetes* were significantly decreased in HSD‐fed mice (Supporting Information: Figures 4F and 5A). It should be noted that an increase in *Bacteroidetes* is considered a normal and healthy gut microbiota state [24], whereas a decrease in *Bacteroidetes* abundance is associated with neurological disorders such as depression, schizophrenia, and autism [25,26]. Further, analysis of the different bacterial abundance at the class level indicated that *Bacilli* was also lower in HSD‐fed mice (Supporting Information: Figures 4G and 5B). We found that HSD‐fed mice displayed different bacterial profiles from NSD‐fed mice, driven by some taxa such as *Staphylococcus* and *Faecalibaculum* (Supporting Information: Figure 5C). These results were further verified by RandomForest classification analysis at the genus level (Supporting Information: Figure 5D), which showed that the bacterial taxa contributed to discrimination between the two groups, mainly included *Dubosiella*, *Faececali baculum*, *Lactobacillus*, *Parasutterella*, and *Butyricimonas*.

Next, we explored specific differentiation at the species levels and identified five significantly altered bacteria. The results showed that the abundance of *B. virosa* and *Lactobacillus johnsonii* was significantly decreased in HSD‐fed mice, the abundance of *Faecalibaculum rodentium*, *Staphylococcus xylosus*, and *Parasutterella excrementihominis* was significantly increased (Supporting Information: Figure 5E–I), and *B. virosa* could metabolize fiber into butyrate [27, 28]. Recently, butyrate has received attention on intestinal homeostasis [29, 30].

SCFAs play an essential role in microbiota‐gut‐brain crosstalk [31]. We also found that the butyrate‐producing bacteria *B. virosa* was significantly decreased in HSD‐fed mice. Hence, we quantified luminal SCFAs in both groups and found that the levels of propionic acid, isobutyric acid, butyric acid, isovaleric acid, and valeric acid were lower in HSD‐fed mice, while there was a similar level of acetic acid and caproic acid displayed in mice fed with HSD or NSD (Supporting Information: Figure 6). Taken together, HSD induced a remarkable alteration of gut microbiota composition and SCFAs production, and the shift had unfavorable effects on memory function.

### Gut microbiota from HSD impairs memory and synapse

**Figure 2:**
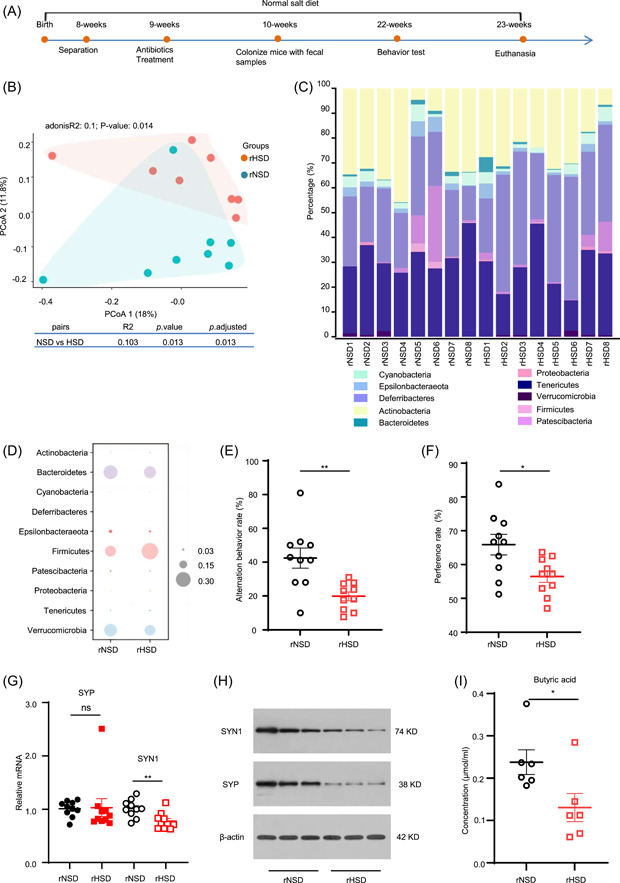
Fecal microbiota from high‐salt diet (HSD)‐fed mice transplanted into the normal salt diet (NSD)‐fed mice elicited synapse loss and memory impairment. (A) Study design. Mice were raised under identical specific pathogen‐free conditions and fed with NSD for 16 weeks. The mice were orally gavaged with an antibiotics cocktail for 1 week and divided into two groups: rHSD group (*n* = 10) and rNSD group (*n* = 10), which were orally gavaged with feces collected from HSD‐ and NSD‐fed mice, respectively. Fecal microbiota transplantation (FMT) was conducted 4 times per week for 3 months and, after that, behavior tests were performed, following which the mice were killed for further analysis. (B) Principal component analysis plot (PCoA) based on sequence abundance of bacterial 16S ribosomal DNA gene in fecal content revealed by Bray–Curtis dissimilarity. (C) Total relative abundance of prevalent microbiota at the phylum level, different categories of gut microbiota were colored differently, and their changes in relative abundance were presented on the barplot. (D) Comparison of relative abundance of prevalent microbiota at the phylum level. (E) Percentage of spontaneous alternations performance in Y‐maze test. (F) Percentage of exploration time during recognition trail in the novel object recognition task. (G) Percentage of exploration during recognition trail in the novel object recognition task (*n* = 10). Changes in the relative messenger RNA (mRNA) expression levels of SYP (H) and SYN1 (I) in the hippocampus of mice (*n* = 10). (J) Changes of the protein levels of SYP and SYN1 in the hippocampus of mice by western blot analysis. (I) The levels of butyric acid measured by gas chromatography–mass spectrometry (GC‐MS) from fecal samples (*n* = 6). Data are expressed as mean ± SEM; *p* values were calculated by the nonparametric Mann–Whitney test; *p < 0.05; **p < 0.01.

Here we investigated whether altered gut microbiota induced by HSD affected cognition. Fecal samples were collected from NSD‐ or HSD‐fed mice, then transplanted into NSD‐fed mice (Figure 2A). When analyzing the fecal microbiota profiles, we observed a significant differential clustering of gut microbiota community between the rNSD mice (mice accepted fecal samples from NSD‐fed mice) and the rHSD mice (mice that were transplanted with fecal samples from HSD‐fed mice), as revealed by principal coordinates analysis (PCoA) (Figure 2B). Further analysis confirmed the differences between the microbiota phylum in both recipient groups (Figure 2C,D), including phylum affected by HSD, such as *Bacteroidetes*. Compared to rNSD mice, the rHSD mice displayed remarkable memory impairment, while no significant result was observed in the Y maze test (Figure 2E,F and Supporting Information: Figure 7G,H).

Further, we examined the expression levels of SYP and SYN1 in both recipient groups and found that compared to rNSD mice, the mRNA and protein expression of SYN1 were decreased in rHSD mice (Figure 2G,H), whereas no significant change was observed in SYP. We also detected SCFAs and the rHSD mice showed a significant decrease in butyric acid (Figure 2I), whereas there were no differences between the other six SCFAs of both recipient groups (Supporting Information: Figure 7A–F). Our results indicated that HSD‐induced microbiota dysbiosis could promote cognitive impairment and synapse loss.

### Microbiota from HSD‐fed mice extensively altered the brain transcriptome

We conducted full‐length transcriptome sequencing of brain samples collected from rNSD and rHSD mice. The two groups displayed similar disruption and variation in total gene expression levels (Supporting Information: Figure 8A,B). However, significant differential clustering of gene expression was observed by principal component analysis (Supporting Information: Figure 8C) and the two groups exhibited different activities of transcription factor (Supporting Information: Figure 8D), which indicated the involvement of HSD‐related microbiota in brain gene expression. Moreover, a total of 489 genes were significantly and differentially (DEGs) expressed between rHSD and rNSD mice, which comprised 197 upregulated and 292 downregulated genes. Heatmap was used to visualize these DEGs (Supporting Information: Figure 9A,B and Table 1). Genes differentially regulated in rHSD mice compared with rNSD mice were subjected to Gene Ontology (GO) and Kyoto Encyclopedia of Genes and Genomes (KEGG) analyses. GO enrichment analysis of DEGs revealed that the synaptic function and composition in rHSD mice were weaker than in rNSD mice. For instance, regarding biological processes, the presynaptic process involved in chemical synaptic transmission was significantly enriched. For cellular components, we noted that the synapse and synapse parts were significantly enriched (Supporting Information: Figure 9C), revealing an extensive decline in the expression of genes regulating synaptic function and composition, which were efficient and essential for memory and learning. Genes differentially regulated in rHSD mice compared with rNSD mice were further subjected to KEGG pathway enrichment analyses, and we observed several significantly different KEGG pathways in the two groups. The PI3K/Akt signaling pathway was significantly dysregulated in rHSD mice brains (Supporting Information: Figure 9D). To further verify the role of PI3K, which was activated by HSD, a Western blot was performed, and the results showed that the PI3K/Akt signaling was enhanced after pretreatment with HSD (Supporting Information: Figure 9E).

### Butyrate partially reverses the memory impairment induced by HSD

**Figure 3:**
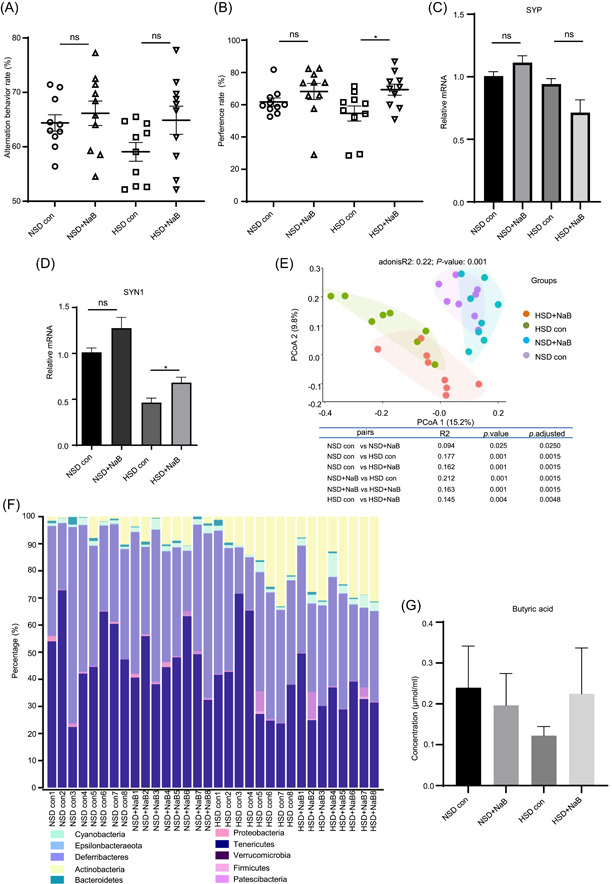
Butyrate partially reverses the high‐salt diet (HSD)‐induced memory impairment and gut microbiota dysbiosis. (A) Percentage of spontaneous alternations performance in the Y‐maze test. (B) Percentage of exploration during recognition trail in the novel object recognition task. Changes in the relative mRNA expression levels of SYP (C) and SYN1 (D) in the hippocampus of mice. (E) Principal component analysis plot (PCoA) based on bacterial 16S ribosomal DNA gene sequence abundance in fecal content was revealed by Bray–Curtis dissimilarity. (F) Total relative abundance of prevalent microbiota at the phylum level, different categories of gut microbiota were colored differently, and their changes in relative abundance were presented on the barplot. (G) The levels of butyric acid were measured by gas chromatography–mass spectrometry (GC‐MS) from fecal samples. *n* = 6–10 per group. The data are expressed as mean ± SEM; *p* values were calculated by the nonparametric Mann–Whitney test, *p < 0.05; **p < 0.01.

We hypothesized that the decreased butyrate production might at least partially explain the cognitive impairment. Therefore, 20 mg/kg of butyrate sodium in drinking water was given for 16 weeks to investigate the possible causal mediator butyrate between HSD and cognition. The results showed that the addition of butyrate partially inhibited the memory impairment induced by the HSD diet (Figure 3A,B and Supporting Information: Figure 10A,B). We further explored the effects of butyrate on synapse and found a significant change in the mRNA expression of SYN1 in HSD‐fed mice receiving butyrate compared with simple HSD‐fed mice (Figure 3C,D). In addition, butyrate supplementation had no significant effect on microbial community composition (Figure 3E,F). Supplementation of HSD‐fed mice with butyrate did not significantly impact the level of butyrate in the colon (Figure 3G) and the other SCFAs (Supporting Information: Figure 10C–H). Our results indicated that butyrate did not cause changes in bacterial composition or increase butyrate levels but could independently restrict the damage of HSD on memory function, which might occur through the regulation of SYN1.

## DISCUSSION

Diet and microbiota composition have been reported to be critical in maintaining cognitive function. Here we showed that a high dietary concentration of NaCl could modulate gut microbiota composition and metabolites by reducing the abundance of butyrate‐producing bacteria spp. and butyrate levels. Furthermore, gut microbiota might be a pivotal mediator in memory function impairment by HSD, because the changes in the gut microbiota of mice fed with HSD were associated with impaired memory function.

Diet is one of the most potent modulators of microbiota composition and function [32]. Our research investigated the association between HSD and gut microbiota. Of note, unlike previous studies on a HSD [33, 34], we did not add salt to the drinking water of the mice, which contributes to a better simulation of unhealthy dietary habits in humans. The results showed that 12 weeks of an HSD significantly affected the gut microbiota and decreased *Bacteroidetes* at the phylum level and *Bacilli* at class level, which were associated with an inflammatory state [35]. Moreover, decreased beneficial *B. virosa* and butyrate contents were associated with healthy brain physiology. Hu et al. [23] found that 12 weeks of HSD significantly reduced the acetate, propionate, and butyrate levels in fecal samples, and Miranda et al. [34] reported that 4 weeks of HSD significantly reduced butyrate concentration. Although different types of intervention and duration of feed exist in these studies, they demonstrated similar consequences on the gut microbiota composition and SCFAs production. Thus, the reduction observed in *B. virosa* might, at least partially, be indirectly responsible for the reduced butyrate levels.

Emerging evidence indicates that HSD might impair cognitive functions [5, 36, 37], and a study showed associations between salt intake and cognitive impairment and performance [38]. Most studies in rodent models found cognitive impairment following high‐salt intake (typically, 4%–8%) diets. Our results suggested that 12 weeks of 8% salt in the diet was sufficient to impair cognitive function, particularly long‐term memory, spatial learning, and memory. However, only a few studies have directly investigated the underlying mechanisms. Another report showed that cognitive dysfunction might be mediated by a deficit in endothelial nitric oxide [39]. Consistent with a previous study [5], our results indicated that HSD affected memory‐related synapses, which induced cognitive impairment. We found that HSD significantly decreased the expression of SYP and SYN1. These molecules are thought to be related to synaptic plasticity [40]. SYP and SYN1 are linked to the release of neurotransmitters, as well as facilitating rapid and efficient neurotransmission [40]. A relationship between memory and SYP‐related proteins was observed in other studies [41, 42], suggesting that dietary salt could affect synaptic‐related memory function.

Syeda et al. [43] indicated that bioactive food could abate synaptic alterations by affecting gut microbiota in Alzheimer's disease (AD) and Buffington et al. [44] found that microbiota reconstitution reversed diet‐induced social deficits in mice. These findings suggest the effects of gut microbiota composition alterations by HSD on synaptic deficits via the gut–brain axis. Herein, we observed that 12 weeks of FMT impaired short‐ and long‐term memory. Notably, mice fed with NSD that accepted FMT from donor mice fed with HSD also showed decreased SYP and SYN1 expression, and the transcriptome in rHSD mice brains were distinct from that in rNSD brains, and the GO pathways of synapse and synapse part were significantly changed in rHSD mice. In addition, gene set enrichment analysis revealed the pathways associated with synaptic structure and function downregulated in rHSD mice brains, such as positive regulation of synaptic transmission, synaptic plasticity, and positive regulation of long‐term potentiation, as well as the structure of the synapse, as the presynaptic membrane. Therefore, the gut microbiota contributed to regulating synaptic structure and function, which could partly explain how HSD impacts memory function.

To verify this interpretation with greater certainty, we evaluated the effects of FMT on the gut microbiota composition and SCFAs production. As expected, the gut microbiota composition of recipient mice appeared to shift towards the pattern of mice fed HSD, as well as the SCFAs production. Recent research suggests an imbalance in gut microbiota‐induced neuropathy by affecting synaptic homeostasis. A study showed that FMT from aged donors caused impaired learning in young recipients. In addition, a reduction of bacteria associated with SCFAs production was observed [45]. Another study showed that gut microbiota affected the brain of healthy mice. The recipient mice showed reduced memory and short‐term object recognition memory [46]. In line with this evidence, we found that gut microbiota from mice fed with HSD had impaired cognitive function, including short‐term object recognition memory and working memory, concomitant with synaptic impairment, indicating a perturbed gut–brain axis.

To further explore the mechanisms underlying synapse loss, we tested the brain transcriptome. KEGG pathway analysis identified alterations in the PI3K/Akt signaling pathway in the brain of rHSD mice, suggesting that the gut microbiota could mediate its change. The PI3K/Akt pathway is essential for initiating immune responses [47, 48]. However, dysregulation of this pathway can lead to detrimental effects, a primary factor in tumorigenesis [49, 50, 51]. Given the known role of inflammatory responses occurring external to the brain [52], dysregulation of the PI3K/Akt signaling may also contribute to the onset and persistence of neuroinflammation, thus suggesting that an over‐activity of the PI3K/Akt signaling could drive the production of pro‐inflammatory cytokines, leading to synaptic loss and cognitive impairment.

The gut microbiota regulates the concentration of several single‐functional or multifunctional signaling molecules in the colon and circulation. We observed altered SCFAs metabolism in mice fed with HSD, especially for butyrate. Meanwhile, FMT from mice fed with HSD to mice fed with NSD also decreased the butyrate content. However, butyrate supplementation improved memory function and synapse‐associated mRNA expression. Butyrate is mainly produced by bacterial fermentation of fibers in the colon, which can also alter gene expression in the brain [31].

Moreover, Jiang et al. [16] found that butyrate supplementation elevated the levels of synapse‐associated proteins and behavioral performance in mice with AD. Consistent with the previous study, our results suggested that a reduction in butyrate production mediated synaptic loss and memory impairment induced by HSD. However, the precise mechanisms remain to be investigated. This could be considered a limitation of our study, which we aim to clarify in our future studies.

Overall, the current research suggests a novel link in the gut‐brain axis via microbial metabolites with HSD, indicating the role of HSD in altering the microbiome and butyrate production that induces synaptic loss and memory impairment. Moreover, our results suggest that dysregulation of the PI3K‐Akt pathway might be an important molecular signal pathway. However, more work is needed to investigate the patterns of HSD in the brain and whether these results are related to the dysregulation of the PI3K‐Akt pathway. Even though the precise mechanisms of action remain uncertain, the present study provides new insights into the mechanisms of excess dietary salt on memory. Furthermore, butyrate was associated with memory improvement and could be potentially considered in treating memory impairment.

## CONCLUSION

HSD affected the relative abundance of butyrate‐producing bacteria (*B. virosa*) and butyrate production, consequently decreasing the expression of synaptic proteins (SYN1) and impairing short‐ and long‐term memory via the PI3K‐Akt pathway.

## MATERIALS AND METHODS

### Animal model

Male C57BL/6 specific pathogen‐free (SPF) mice (6‐week‐old, 18–20 g) were purchased from the Guangdong Province Medical Laboratory Animal Center and mice were housed together (four to five mice per cage) in an SPF environment with a suitable temperature (22°C ± 2°C), humidity (45%–60%) keeping 12 h of light every 24 h, and food and water available ad libitum. To verify the possible causal mediator between HSD and cognition, 20 mg/kg Butyrate sodium was added to the drinking water and given for 16 weeks. The drinking water was refreshed every 2 days. Mice were then divided into four groups and fed for 16 weeks as follows: normal chow (NSD, 0.2% salt, Research Diets Inc., 1001); HSD (8% salt, Research Diets Inc., custom feed); NSD with 20 mg/kg Butyrate sodium (S817488, Macklin) in drinking water (NSD + NaB); HSD with 20 mg/kg NaB (S817488, Macklin) in drinking water (HSD + NaB). All experiments were examined and approved by the Animal Ethics Committee for the Care and Use of Animals of the Guangzhou Medical University. Mice fecal samples were collected 3 days after finishing the above treatment in the morning.

For the FMT experiment, donor mice were fed with NSD or HSD (*n* = 8 in each group) for 16 weeks and recipient mice (*n* = 10 in each group) were fed with NSD for 16 weeks. An antibiotic mixture was given through oral gavage for 1 week to deplete the microbiota. The depletion of gut microbiota was performed as previously described [53]. The mice were given a cocktail of vancomycin (50 mg/kg), neomycin (100 mg/kg), metronidazole (100 mg/kg), and ampicillin (100 mg/kg) once daily by oral gavage for 7 days. The antibiotic powder was dissolved in ultrapure water daily, with an oral gavage volume of 10 μL/g. Then, 24 h after the final gavage of antibiotics, FMT was performed by oral gavage (10 μL/g) for 12 weeks (five times per week, from Monday to Friday). FMT was performed according to the methods described in a previous study [54]. Briefly, for donor mice, we grabbed the mice in the morning and gently slid the mice's abdominal with forceps to promote defecation, collected their feces from the colon, which were put in a 1.5 ml centrifuge tube immediately, followed by the addition of 0.5 ml saline into the pipe and mixing with a vortex finder. They were then centrifuged at a speed of 2000*g* for 5 min, after which saline was added after the supernatant liquid was removed and the filtered liquid was collected after filtering through a straw. The liquid was given to the recipient mice by oral gavage. Three days after finishing, the diet treatment and FMT samples were collected for analysis the following morning.

### Behavioral testing in mice

Behavioral testing was conducted 3 days after finishing the diet pretreatment or FMT. The Y‐maze test for evaluating spatial working memory and reference memory (short‐term memory) was performed [55] and the test was conducted in a Y‐maze consisting of three arms (40 × 10 × 15 cm) and at an angle of 120 to each other, with different geometric shapes attached to each arm. The mice were first placed in a fixed position on one of the arms and allowed to explore the arms freely for 10 min. Then, the mice were returned to their cages. After 1 h, the mice were placed in the same place and allowed to move freely in the three arms for 5 min. When all their limbs were within an arm, this was considered a successful entrance of the mice into the corresponding arm. One alternation defines as access to all three arms consecutively. The arm entries and alternations were recorded (spontaneous alteration/(number of arm entries −2) × 100%). A high percentage was considered to be good working memory, as it indicated that the mouse had recalled the arm it had accessed [55]

The novel object recognition task for evaluating long‐term memory was performed according to a previously published study [56, 57], The novel object recognition task was performed, and the mice were first given a habituation test of 5 min in an open area without any objects, followed by a test phase consisting of two trials starting 24 h later. For the first trial, two objects were placed in parallel in the open field, and the mice were allowed to explore them for 5 min (sample phase). By the end of this phase, the mice returned to their respective cages with a delay of 15 min (±15 s). During the testing, one of the objects was replaced with a new one, and the mice were again placed in the field for another 5 min trial. For both samples and testing phases, exploration was defined as an active event in which the rat was pawing at, sniffing, or whisking with its snout directed at the object from a distance of ~1 cm. Sitting on or next to an object was not counted as active exploration [56] and, during both sample and testing phases, we used manual timers to score the time the mice spent exploring (sniffing or touching) the objects. The experimenters' score exploration time was blinded to the intervention of the animals. The objects used were small toys of similar size cleaned with 70% ethanol before each training period. The percentage of time spent exploring a novel object was calculated with the following formula: time exploring novel object/(time exploring novel object + time exploring familiar object) × 100% [57]. Morris water maze was conducted and performed as described [23]. A probe trial was conducted on day 6; the hidden platform was removed, and the mice were placed in the quadrant opposite the target quadrant and allowed to swim for 90 s. The number of platform crossings was scored.

### Real‐time quantitative PCR (RT‐qPCR)

Three days after finishing the pretreatment of diet or FMT, the mice were anesthetized by intraperitoneal injection of pentobarbital sodium (50 mg/kg) and the brain was separated and quick‐frozen at −20°C for 5 min. Then, the hippocampus was isolated and stored at −80°C. Total RNA was isolated from the dissected tissues using a TRIZOL RNA isolation protocol (R0016, Beyotime Biotechnology), complementary DNA (cDNA) was generated via PrimeScript™ RT reagent (9109, TAKARA), RT‐qPCR was performed with TB Green Premix Ex Taq™ II (RR820A, TAKARA) on a Thermo 7300 using primers derived from IGE Biotechnology for the indicated target genes and quantified as ΔΔC_T_, relative to β‐actin. The 2^‐ΔΔCT^ method was used [58] and all reactions were set with two multiple wells. SYP and Syn1 were detected by PCR. The primers are shown in Supporting Information: Table 2.

### Western blot analysis

Proteins were separated using sodium dodecyl sulfate‐polyacrylamide gel electrophoresis gels and polyvinylidene difluoride membranes (Millipore) with a wet electroblotter (Bio‐Rad) for 120 min at 100 V [59]. The membrane was incubated with the primary antibody overnight at 4°C and then incubated for 1 h with the secondary antibody in Tris‐Buffered Saline, 0.1% Tween 20 buffer for 4 h at 4°C. The membrane was developed by the enhanced chemiluminescence method (ECL kit; Pierce) [60].

### SCFAs quantification

Fecal SCFAs concentration was determined by WEKEMO Technologies Co, Ltd. Briefly, the feces (~100 mg) were thawed and suspended, followed by the addition of 50 μL 15% phosphoric acid and 100 μL of 125 μg/mL internal standard (isohexic acid) solution, and ether 400 μL to homogenate (Multitube mixer BE‐2600) for 1 min, and then centrifuged at 12,000 r.p.m. for 10 min. The supernatant was filtered before GC‐MS analysis (Thermo Trace 1300). The injected sample volume for GC‐MS analysis was 1 µL.

### Microbiome 16S rDNA sequencing and data analysis

Bacterial DNA was extracted from fecal samples using the QIAamp DNA stool Mini Kit (Qiagen). The sequencing library was constructed, then paired‐end sequenced (2 × 250 bp) on an Illumina MiSeq platform at Biomarker Technologies Co, Ltd.

The QIIME2 software was used to analyze the gut microbiota α diversity. The α diversity indicators included the Chao1 index, Ace index, Shannon index, and Simpson index. β‐Diversity was analyzed by presenting PCoA based on the Bray–Curtis dissimilarity, Jaccard distances, and NMDS. Metastats software was used to compare the relative abundance of bacteria at different levels between the two groups, and the *p* value was obtained by nonparametric tests. By correcting the *p* value to access the *q* value, the species responsible for the difference in sample composition between the two groups were screened according to *p* values (or *q* values), with a default *p* ≥ 0.05. LefSe was used to compare the relative abundance of the bacteria at the phylum, class, and genus levels between the two groups. Linear discriminant analysis (LDA) was used to estimate the magnitude of the effect, setting the logarithmic LDA score for significant differences at 2.0. This analysis primarily intended to find species that differed significantly in abundance between groups.

### Transcriptome sequencing and data analysis

The cDNA libraries were sequenced on the Illumina HiSeqX‐tenten platform. Briefly, 1 μg total RNA was used to prepare cDNA libraries using the cDNA‐PCR sequencing kit (SQK‐LSK110 + EXP‐PCB096) using the protocol of Oxford NanoporeTechnologies. The reverse‐transcriptase template switching activity caused the enrichment of the full‐length cDNAs and the addition of defined PCR adapters to the ends of the first strand cDNA directly. The cDNA was further amplified for 14 circles with LongAmp Tag (NEB). The ONT aptamer ligation of the PCR products was then performed using T4 DNA ligase (NEB). Agencourt XP beads were used for DNA purification based on the ONT protocol. The final cDNA library was added to the FLO‐MIN109 flow cells and operated on the PromethION platform at the Biomarker Technology Company. The data were analyzed for differential expression using the edgeR package (3.8.6) for identifying the differential in the numeric gene expression data using a model based on a negative binomial distribution. *p* values were adjusted to control for false discovery rates using Benjamini and Hochberg's approach. Lastly, the genes identified by edgeR with *p* < 0.05 and fold changes ≥1.5 were designated as differentially expressed.

The statistical enrichment of differential expression genes in the KEGG pathways was tested by the KOBAS software (http://www.genome.jp/kegg/) [61].

### Statistical analyses

SPSS v25.0 software (IBM Corp.) was used. Normally distributed variables were analyzed using the Student's *t* test, and non‐normally distributed variables using an unpaired two‐tailed *t* test (Mann–Whitney *U* test). A *p* < 0.05 was considered significant for comparison between groups.

## AUTHOR CONTRIBUTIONS

Chao Lei, TingLiu, and ZhihuaLiu contributed to the conception and design of the study. Chao Lei and Cong Liu performed the data analysis and interpretation. Chao Lei wrote the first draft of the manuscript and Ting Liu revised the manuscript. Chao Lei, Cong Liu, Yu Zhan, and Yuling Peng conducted the experiments. Chao Lei, Cong Liu, Yuling Peng, and Xiaoming Zhang participated in analyzing and interpreting the data. All authors contributed to the article and approved the submitted version.

## CONFLICT OF INTEREST STATEMENT

The authors declare no conflict of interest.

## ETHICS STATEMENT

The studies were reviewed and approved by The Guangzhou Medical University Animal Ethics Committee (2018‐083) for the present study.

## Supporting information

Supporting Information.

Supporting Information.

Supporting Information.

## Data Availability

The data sets generated during the current study can be downloaded from Sequence Read Archive with Accession number: PRJNA863766 (http://www.ncbi.nlm.nih.gov/bioproject/863766). The data sets during the current study are available from the corresponding author on request.
