# Supplementary material for: A high‐salt diet induces synaptic loss and memory impairment via gut microbiota and butyrate in mice
Source: Imeta. 2023 Mar 21;2(2):e97. doi: 10.1002/imt2.97 (PMC10989808; doi:10.1002/imt2.97)
Supplement: Supplementary file 2 — Supporting Information. [file IMT2-2-e97-s001.docx]

**Table S2 Primers used in the Real-time PCR in our sutdy**

| Gene | forward primer | reverse primer |
| --- | --- | --- |
| β-actin  SYP  SYN1 | GCTTCTTTGCAGCTCCTTCGT  TACCGAGAGAACAACAAAGGGC  ACCGACTGGGCAAAATACTTCA | GCTTTGCACATGCCGGA  ACTAGCCACATGAAAGCGAACA  CCATGTCCACAGAGAATCCACC |
