# Supplementary material for: A high‐salt diet induces synaptic loss and memory impairment via gut microbiota and butyrate in mice
Source: Imeta. 2023 Mar 21;2(2):e97. doi: 10.1002/imt2.97 (PMC10989808; doi:10.1002/imt2.97)
Supplement: Supplementary file 3 — Supporting Information. [file IMT2-2-e97-s002.docx]

**High salt diet induces synaptic loss and memory impairment via gut microbiota and butyrate in mice**

Running title: High salt diet and memory impairment

**Chao Lei****^1,2†^, Cong Liu^2†^, Yuling Peng^2†^, Yu Zhan^2^, Xiaoming Zhang^3^, Ting Liu^2,*^, Zhihua Liu^1,2 *^**

^1^ Department of Anorectal Surgery, Affiliated Dongguan Hospital, Southern Medical University (Dongguan People's Hospital), Dongguan, China;

^2^ Innovation Centre for Advanced Interdisciplinary Medicine, Key Laboratory of Biological Targeting Diagnosis, Therapy and Rehabilitation of Guangdong Higher Education Institutes, the Fifth Affiliated Hospital of Guangzhou Medical University, Guangzhou 123456, China;

^3^ Department of Internal Medicine, Huazhong University of Science and Technology Union Shenzhen Hospital, Shenzhen 123456, China;

**Supporting Information**

Supplementary FIGURE 1. The body weight, food, water, salt intake, and length of the small intestine and colon of mice a-f, Body weight (A), food intake (B), length of the small intestine (C) and colon (D), water intake (E), and salt intake (F) of mice. n =15 per group; data are shown as mean±s.e.m; p values were calculated by two-tailed Student’s t-test, ***, P<0.001.

Supplementary FIGURE 2. The effect of HSD on the locomotion of mice in Y-maze test and novel object recognition task. (A) The total number of entries in the Y-maze task. (B) Total exploration time during the recognition trial in the novel object recognition task. n =16 per group. Data are expressed as the mean±s.e.m; p values were calculated by the non-parametric Mann-Whitney test.

Supplementary FIGURE 3. Effects of the high-salt diet (HSD) on the long-term memory ability of mice using the Morris water maze. (A) Number of platform crossings during the probe trial. (B) The percentage of the time spent in the SW quadrant (%) for the mice in the control and HSD groups. n =10 in each group. Data are expressed as mean±s.e.m; p-values were calculated by the non-parametric Mann-Whitney test, *, p < 0.05, **, p < 0.01.

Supplementary FIGURE 4. HSD altered the gut microbiota structure. (A) Scheme representing the microbiome profiling experiment in mice fed NSD or HSD. Comparison of alpha-diversity of gut microbiota among the two groups of mice using Shannon’s index (diversity) (B) and the Chao1 index (richness) (C). Principal component analysis plot (PCoA) based on bacterial 16S ribosomal DNA gene sequence abundance in fecal content as revealed by Jaccard distances (D), Non-Metric Multi-Dimensional Scaling(NMDS) analysis (E). Total relative abundance of prevalent microbiota at the phylum (F) and class (G) level in the two groups. The data for individual mice are shown, with different categories of gut microbiota colored differently, and their changes in relative abundance are presented on the barplot. n =8 per group.

Supplementary FIGURE 5. HSD alters the gut microbiota profiles. (n =8 per group) (A) Relative abundance of Bacteroidetes. (B) Relative abundance of Bacilli. (C) LEfSe analysis of taxa significantly enriched in NSD-fed mice (green) or HSD-fed mice (red). (D) RandomForest analysis of microbiome features of NSD-fed and HSD-fed mice. (E) Relative abundance of *Butyricimonas virosa*. (F) Relative abundance of Lactobacillus johnsonii. (G) Relative abundance of Parasutterella excrementihominis. (H) Relative abundance of Faecalibaculum rodentium, (I) Relative abundance of Staphylococcus xylosus. Data are expressed as mean±s.e.m; p values were calculated by the non-parametric Mann-Whitney test, *, p < 0.05; **, p < 0.01.

Supplementary FIGURE 6. HSD alters SCFAs production. The levels of SCFAs measured by GC-MS from fecal samples. The isobutyric acid (A), isovaleric acid (C), and valeric acid (D) were lower in mice fed with HSD, while the acetic acid (B) and caproic acid (E) displayed similar levels in mice fed with HSD or NSD. n =12 per group. Data are expressed as mean±s.e.m; p-values were calculated by the non-parametric Mann-Whitney test, *p < 0.05, **p < 0.01.

Supplementary FIGURE 7. SCFAs quantification of fecal samples and behavior tests in rNSD and rHSD mice. The level of acetic acid (A), propionic acid (B), isobutyric acid (C), valeric acid (D), isovaleric acid (E), and caproic acid (F) in fecal samples measured by GC-MS. (G) The total number of entries in the Y-maze test. (H) The total exploration time during the recognition trial in the novel object recognition task. n =6-15 per group. Data are expressed as mean±s.e.m; p values were calculated by the non-parametric Mann-Whitney test.

Supplementary FIGURE 8. Full-length transcriptome sequencing of brain samples collected from rNSD mice and rHSD mice. Disruption(A) and variation (B) of total gene expression level. (C) Principal component analysis (PCA) showing the differential clustering of gene expression between rNSD brains and rHSD brains, (D) Heatmap of significantly different activity of transcription factor. Red areas represent high expression, and green areas represent low expression. n =8 per group.

Supplementary FIGURE 9. Identification of DEGs for enrichment analysis between the rNSD and rHSD mice. (A) Heatmap of DEGs between the rNSD and rHSD samples. Red areas represent high expression, and green areas represent low expression. (B) Volcano plots of the significantly differentially expressed genes. Fold-change (X-axis) and p-value (Y-axis) of DEGs comparing rHSD group versus rNSD group. Each dot represents a single DEG, and the dashed line on the Y-axis illustrates statistical significance (p =0.05), green-downregulated, red-upregulated. (C) GO pathway enrichment analyses of DEGs. The bar graph shows the top 23 (red for biological processes), the top 17 (blue for molecular function), and the top 18 (green for the cellular component) enriched GO pathways. (D) KEGG pathways-related genes were significantly upregulated and downregulated in the brains of rHSD versus rNSD mice by statistics of pathway enrichment. (E) Western blot showed that the P-PI3K signaling was enhanced after the pretreatment of HSD. n =8 per group.

Supplementary FIGURE 10. The effect of butyrate on memory in mice fed with HSD. (A) The total number of entries in the Y-maze task. (B) The total exploration time during the recognition trail in the novel object recognition task. The level of acetic acid (C), propionic acid (D), isobutyric acid (E), isovaleric acid (F), valeric acid (G), and caproic acid (H) in fecal samples measured by GC-MS. n =6-10 per group; data are expressed as mean±s.e.m; p values were calculated by the nonparametric Mann-Whitney test.
